# Reintervention technique for endoscopic ultrasound-guided hepaticogastrostomy combined with bridging stent deployment using a novel double-lumen dilator

**DOI:** 10.1055/a-2545-8043

**Published:** 2025-03-12

**Authors:** Nobu Nishioka, Takeshi Ogura, Yuki Uba, Takafumi Kanadani, Hiroki Nishikawa

**Affiliations:** 1 2nd Department of Internal Medicine, Osaka Medical and Pharmaceutical University, Takatsuki, Japan; 2 Endoscopy Center, Osaka Medical and Pharmaceutical University Hospital, Takatsuki, Japan

Endoscopic ultrasound (EUS)-guided hepaticogastrostomy (HGS) can be indicated for failed endoscopic retrograde cholangiopancreatography (ERCP). In cases of hilar biliary obstruction (HBO), the use of a bridging technique under EUS guidance has been reported 1 2 3 . However, reintervention for HBO after the bridging technique may be challenging because of complex uncovered self-expandable metal stent (UCSEMS) deployment. Recently, a novel dedicated double-lumen dilation device (Meissa; Japan Life Line, Tokyo, Japan) for dilation of the tract during EUS-HGS has become available ( Fig. 1 ). The device has a 2.3-Fr tip and a maximum diameter of 7.4 Fr. There is also a side hole 2 cm from the tip. Contrast medium injection, aspiration of bile juice, and 0.025-inch guidewire insertion can be performed. Therefore, implementing a double-guidewire technique is possible using this device. Successful reintervention for recurrent biliary obstruction after EUS-HGS with bridging technique is described.

**Fig. 1:**
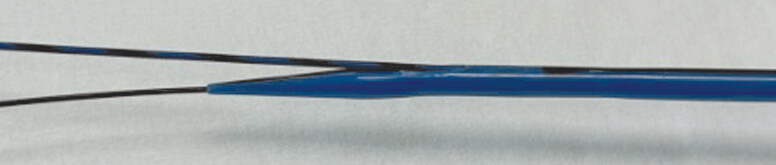
A novel dedicated double-lumen dilation device (Meissa; Japan Life Line, Tokyo, Japan).

A 79-year-old man was admitted to our hospital due to obstructive jaundice after EUS-HGS with bridging UCSEMS deployment. Reintervention was attempted. First, an ERCP catheter was inserted through the mesh of the EUS-HGS stent. The guidewire was successfully inserted into the common bile duct, but the ERCP catheter could not be inserted through the mesh of the UCSEMS. Then, insertion of the novel double-lumen dilator into the common bile duct was attempted and successfully performed. Next, the dilator was pulled back within the UCSEMS, and a guidewire was also inserted through the lumen of the device into the posterior bile duct ( Fig. 2 ). The novel dilator was inserted again along this guidewire, and the guidewire was easily deployed into the anterior bile duct because the lumen of the posterior bile duct was occluded by the dilator ( Fig. 3 ). Subsequently, the UCSEMS was successfully deployed in the anterior bile duct ( Fig. 4 ). Next, the guidewire was inserted into the posterior bile duct through the mesh of this UCSEMS. The UCSEMS was then deployed within an obstructed UCSEMS using the stent-in-stent technique ( Fig. 5 ). Finally, a UCSEMS was deployed from the left bile duct to the stomach without any adverse events ( Video 1 ).

**Fig. 2:**
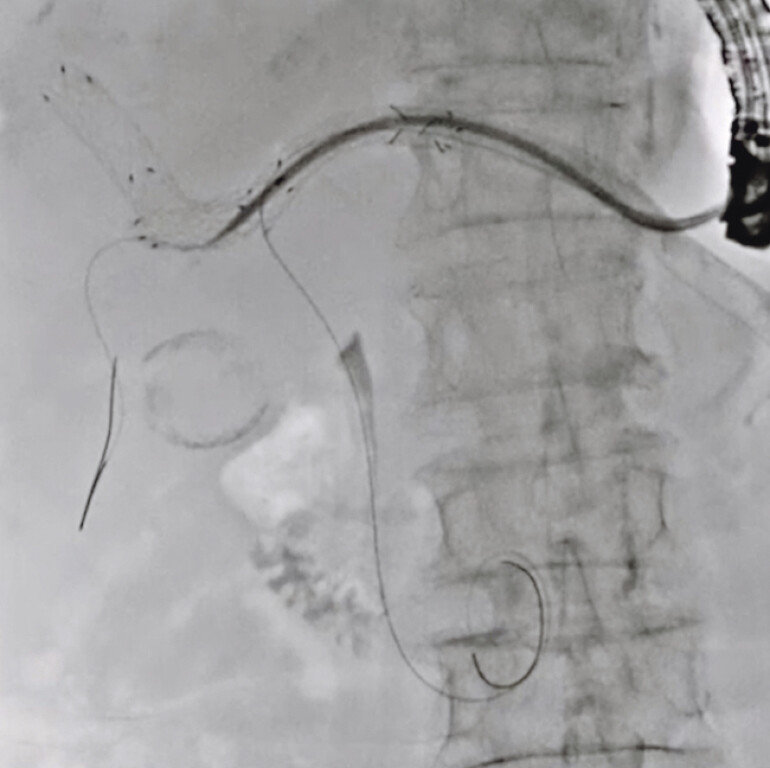
A guidewire was inserted through the lumen of the device into the posterior bile duct.

**Fig. 3:**
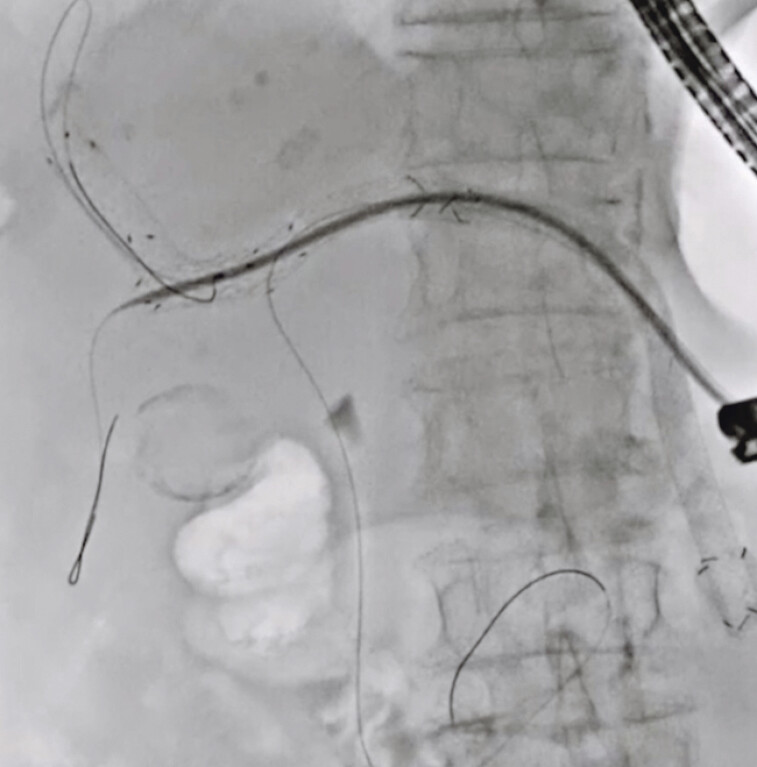
The guidewire was easily deployed into the anterior bile duct because the lumen of the posterior bile duct was occluded by the dilator.

**Fig. 4:**
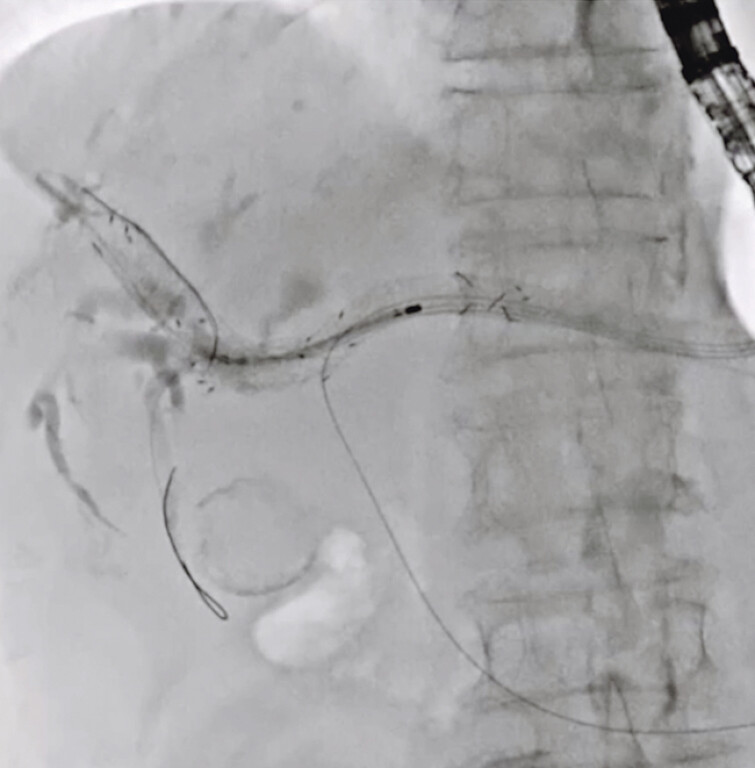
An uncovered self-expandable metal stent was successfully deployed in the anterior bile duct.

**Fig. 5:**
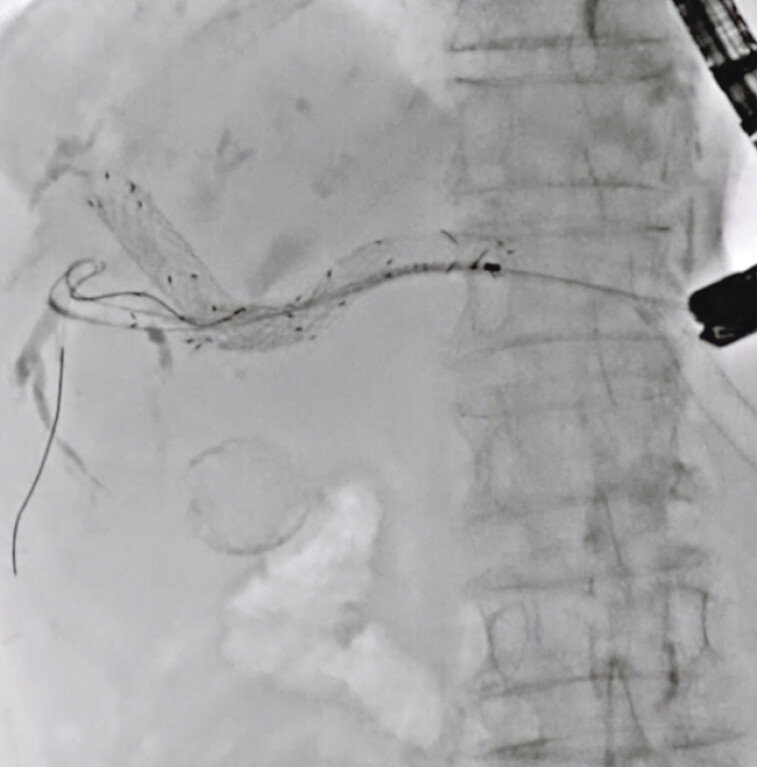
The uncovered self-expandable metal stent was deployed within an obstructed uncovered self-expandable metal stent using the stent-in-stent technique.

Reintervention technique for endoscopic ultrasound-guided hepaticogastrostomy combined with bridging stent deployment using a novel double-lumen dilator.Video 1

In conclusion, this device can be used as a dilation device not only during EUS-HGS, but also during reintervention for an occluded UCSEMS.

Endoscopy_UCTN_Code_TTT_1AS_2AH

## Notes

Endoscopy E-Videos https://eref.thieme.de/e-videos
*E-Videos* is an open access online section of the journal *Endoscopy* , reporting on interesting cases and new techniques in gastroenterological endoscopy. All papers include a high-quality video and are published with a Creative Commons CC-BY license. Endoscopy E-Videos qualify for HINARI discounts and waivers and eligibility is automatically checked during the submission process. We grant 100% waivers to articles whose corresponding authors are based in Group A countries and 50% waivers to those who are based in Group B countries as classified by Research4Life (see: https://www.research4life.org/access/eligibility/ ). This section has its own submission website at https://mc.manuscriptcentral.com/e-videos .
